# Human T-Lymphotropic Virus type 1 infection in an Indigenous Australian population: epidemiological insights from a hospital-based cohort study

**DOI:** 10.1186/s12889-016-3366-5

**Published:** 2016-08-15

**Authors:** Lloyd Einsiedel, Richard J. Woodman, Maria Flynn, Kim Wilson, Olivier Cassar, Antoine Gessain

**Affiliations:** 1 Flinders University/Northern Territory Rural Clinical School, Alice Springs Hospital, Rubuntja Building, 0870 Northern Territory, Australia; 2 National Serological Reference Laboratory, Melbourne, Australia; 3 Institut Pasteur, Unité d’Epidémiologie et Physiopathologie des Virus Oncogènes, Département de Virologie, F-75015 Paris, France; 4 CNRS, UMR 3569, 28 Rue du Dr. Roux, F-75015 Paris, France; 5 Aboriginal Health Unit, BakerIDI,central Australia, Alice Springs Hospital, 0870 Northern Territory, Australia

**Keywords:** HTLV-1, Sexually transmitted infections, Epidemiology, Indigenous, Australia

## Abstract

**Background:**

The Human T Lymphotropic Virus type 1 (HTLV-1) subtype C is endemic to central Australia where each of the major sequelae of HTLV-1 infection has been documented in the socially disadvantaged Indigenous population. Nevertheless, available epidemiological information relating to HTLV-1c infection is very limited, risk factors for transmission are unknown and no coordinated program has been implemented to reduce transmission among Indigenous Australians. Identifying risk factors for HTLV-1 infection is essential to direct strategies that could control HTLV-1 transmission.

**Methods:**

Risk factors for HTLV-1 infection were retrospectively determined for a cohort of Indigenous Australians who were tested for HTLV-1 at Alice Springs Hospital (ASH), 1st January 2000 to 30th June 2013. Demographic details were obtained from the ASH patient management database and the results of tests for sexually transmitted infections (STI) were obtained from the ASH pathology database.

**Results:**

Among 1889 Indigenous patients whose HTLV-1 serostatus was known, 635 (33.6 %) were HTLV-1 Western blot positive. Only one of 77 (1.3 %) children tested was HTLV-1 infected. Thereafter, rates progressively increased with age (15–29 years, 17.3 %; 30–49 years, 36.2 %; 50–64 years, 41.7 %) reaching 48.5 % among men aged 50–64 years. In a multivariable model, increasing age (OR, 1.04; 95 % CI, 1.03–1.04), male gender (OR, 1.41; 95 % CI, 1.08–1.85), residence in the south (OR, 10.7; 95 % CI, 7.4–15.6) or west (OR, 4.4; 95 % CI, 3.1–6.3) of central Australia and previous STI (OR, 1.42; 95 % CI, 1.04–1.95) were associated with HTLV-1 infection. Infection was acquired by three of 351 adults who were tested more than once during the study period (seroconversion rate, 0.24 (95 % CI = 0.18–2.48) per 100 person-years).

**Conclusions:**

This study confirms that HTLV-1 is highly endemic to central Australia. Although childhood infection was documented, HTLV-1 infection in adults was closely associated with increasing age, male gender and STI history. Multiple modes of transmission are therefore likely to contribute to high rates of HTLV-1 infection in the Indigenous Australian population. Future strategies to control HTLV-1 transmission in this population require careful community engagement, cultural understanding and Indigenous leadership.

## Background

The Human T Lymphotropic Virus type 1 (HTLV-1) is an oncogenic retrovirus that preferentially infects CD4+ T cells [1]. At least 5–10 million HTLV-1 infected people reside in clusters of high endemicity worldwide [2]. One such endemic focus is present in central Australia where infection with the Australo-Melanesian HTLV-1 subtype C is prevalent [3]. In less than 10 % of cases, HTLV-1 infection is complicated by sequelae that include a rapidly progressive Adult T cell Leukemia/Lymphoma (ATLL) [1, 4] and inflammatory disorders, such as HTLV-1 associated myelopathy/tropical spastic paraparesis (HAM/TSP) [1]. In resource poor areas, infection with other pathogens also contribute to HTLV-1 related morbidity and mortality [5]. The virus is closely cell associated and transmission typically follows exposure to infected lymphocytes in blood, through breast-feeding or sexual intercourse [1]. Infection rates generally increase with age, particularly among women who are thought to be at greatest risk of sexual transmission [1].

**Fig. 1:**
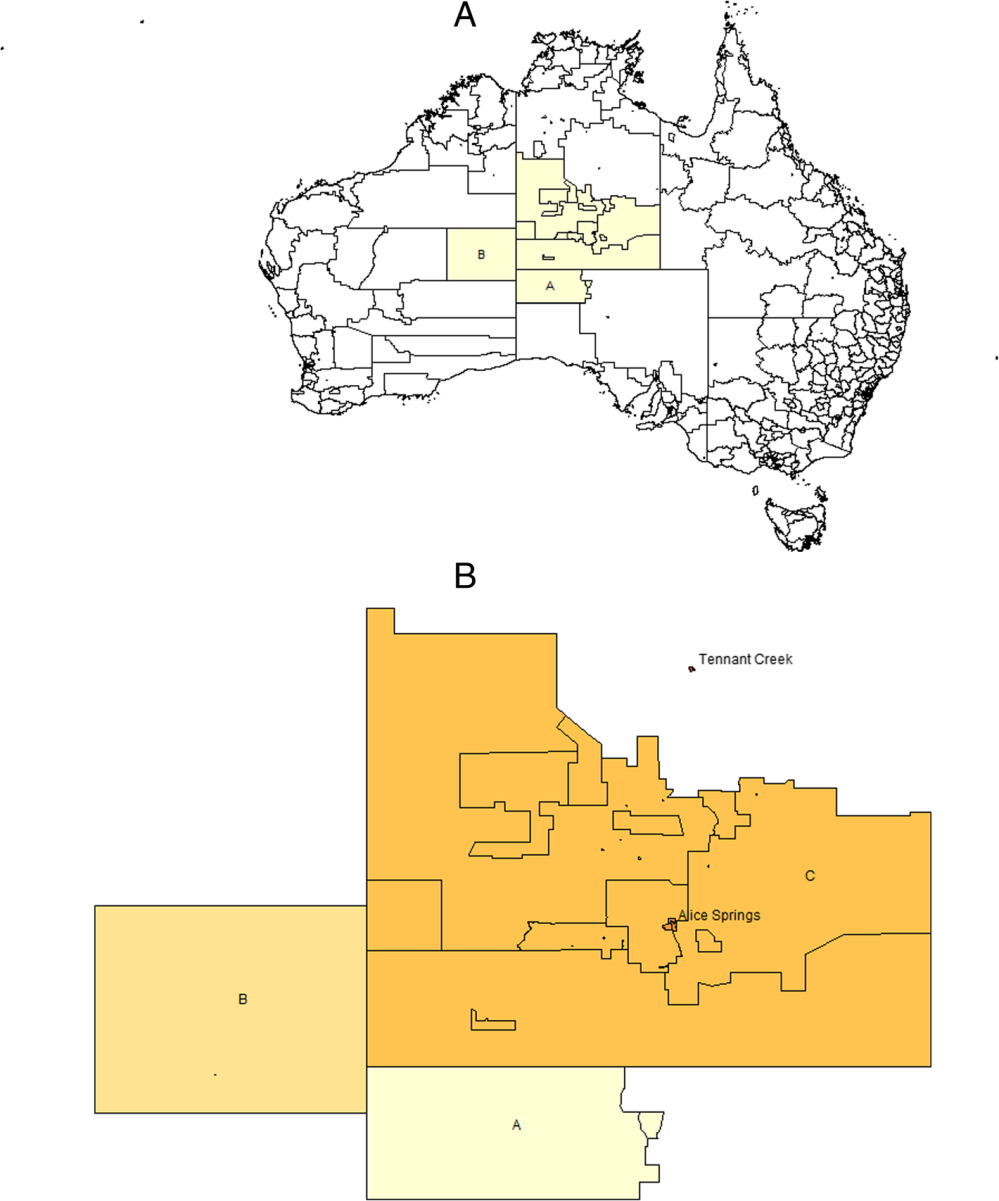
**a**. Map showing the area served by Alice Springs Hospital (*yellow*), which includes the Anangu Pitjantjatjara Yankunyatjara (APY) lands of South Australia (A), the Ngaanyatjarraku shire of West Australia (B) and the Central Desert Shire and MacDonnell Shires of the Northern Territory (C). **b**. Detailed map of central Australia showing the location of the two major population centres, Tenant Creek and Alice Springs

HTLV-1 was first found to be endemic to central Australia in 1988 [6] and this was later shown to be a unique variant of HTLV-1 subtype C [3]. Each of the major recognised complications of HTLV-1 infection have now been reported from this region [4, 5]. These include ATLL [4], HAM/TSP [5, 7], infective dermatitis [8], strongyloidiasis [9], HTLV-1 associated pulmonary disease [5, 10] and crusted scabies [11, 12]. Nevertheless, no coordinated program has been implemented to inform Indigenous Australians of the risks posed by HTLV-1 infection or to prevent viral transmission in this population and HTLV-1 testing is not included in routine antenatal screening [13]. The development of strategies to control HTLV-1 transmission in remote Australia is hampered by limited epidemiological data. Determining HTLV-1 seroprevalence rates in the small communities that are scattered across central Australia, an area exceeding 1 million km^2^ (Fig. 1), is logistically difficult. Indeed, published data are currently available from only two small community-based studies that included 36 [14] and 131 [6] central Australian subjects and neither study was designed to identify risk factors for HTLV-1 transmission. In endemic areas, such as south-western Japan, mother-to-child transmission has been demonstrated to be the primary mode of transmission [15] and this is assumed to be the case in Australia [16]. The present study was therefore commenced to provide some insights into the epidemiology of HTLV-1c infection in central Australia from a large hospital-based cohort. Our analysis strengthens previous findings regarding seropositivity rates according to place of residence [5, 16], defines risk according to age and gender and identifies possible risk factors for horizontal transmission.

## Methods

### Study setting

**Fig. 2:**
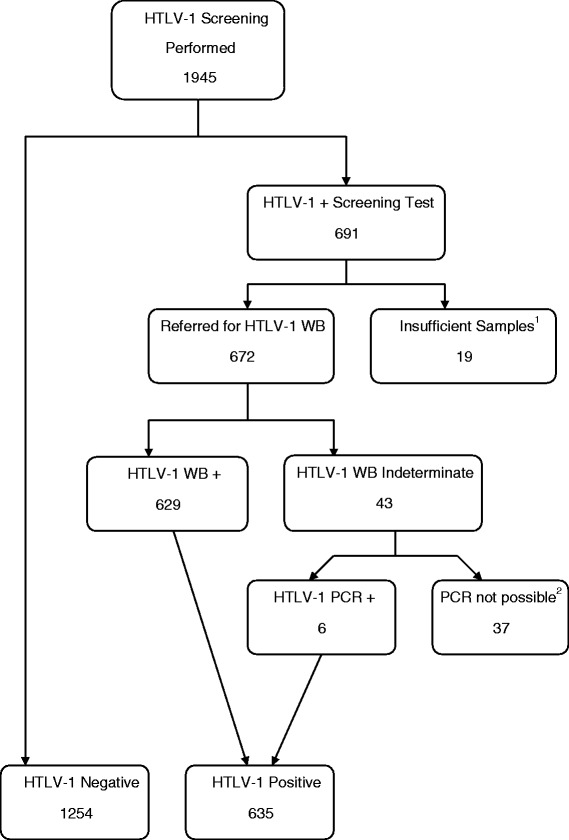
Flow diagram showing reasons for excluding patients from analysis. 1945 subjects were initially screened for HTLV-1 infection using serological tests. In 56 cases (Group 1, 48; Group 2, 4; Group 3, 4), initial serological screening tests were positive, but subjects were excluded because confirmatory testing could not be performed. In 19 cases confirmatory Western blot tests could not be performed because insufficient sample remained after the initial screening tests. In a further 37 cases, Western blots were indeterminate, but an appropriate sample for HTLV-1 PCR could not be collected because patients had returned to their remote communities before the Western blot result was available to clinicians. Thus, 1889 subjects were included in the final analysis. Abbreviations: HTLV-1+, positive HTLV-1 serological screening test; HTLV-1 WB+, positive HTLV-1 Western blot test; PCR, polymerase chain reaction; WB, Western blot

All Indigenous children (aged <15 years) and adults (aged ≥15 years) who attended Alice Springs Hospital (ASH) between 1^st^January 2000 and 30th June 2013 who had an HTLV-1 screening test performed were identified from hospital pathology records. Among 1945 subjects for whom a screening test was performed, HTLV-1 serostatus could be confirmed for 1889 subjects and these were included in the final epidemiological analysis (see flowchart; Fig. 2). The cohort includes patients who were tested: i) when clinically indicated to investigate the aetiology of conditions that are thought to be HTLV-1 associated (Group 1, *n* = 1431), ii) as part of a blood borne virus (BBV) surveillance program among patients receiving haemodialysis (Group 2, *n* = 334) and iii) after enrollment as subjects without current clinical evidence of HTLV-1 associated conditions in HTLV-1 pathogenesis studies (Group 3, *n* = 124). Conditions that are associated with HTLV-1 infection for which HTLV-1 serology is ordered at ASH include haematological malignancies, neurological disorders, strongyloidiasis, chronic lung disease and dermatological conditions. However, the test is most often ordered to accompany strongyloides serology due to concerns that HTLV-1 coinfected patients are more likely to fail therapy and to develop complicated strongyloidiasis [5]. Demographic data including ethnicity, place of residence and dates of birth and death were obtained from the ASH patient management system. Results of tests for sexually transmitted infections (STIs) were recorded from the hospital pathology database for adults aged 15–45 years for whom such tests are routinely ordered at ASH in the setting of genitourinary symptoms. These included nucleic acid amplification tests (NAAT) for *Chlamydia trachomatis* and *Neisseria gonorrhoeae* and specific tests for syphilis (fluorescent treponemal antibody tests and *Treponema pallidum* particle agglutination tests).

### Residence

Place of residence was categorized as i) remote (>80 km from Alice Springs), ii) Alice Springs town camp and iii) urban (resident in Alice Springs, but not in a town camp). Remote residence was further divided into quadrants (north, south, east and west) relative to Alice Springs. Central Australian residence was defined as residence in the Alice Springs Municipality, Central Desert Shire and MacDonnell Shires of the Northern Territory, the Ngaanyatjarraku shire of Western Australia and the Anangu Pitjantjatjara Yankunyatjara (APY) lands of South Australia (Fig. 1).

### Estimating the number of infants at risk

The number of live infants born to Indigenous mothers at ASH for the years 2010–12, the dates of birth and place of maternal residence were obtained from the ASH patient management database. An estimate of the number of infants at risk of mother-to-child HTLV-1 exposure was then calculated by multiplying the total number of infants born to mothers from each area by HTLV-1 seropositivity rates for women aged 15–40 years who resided in the same region.

### HTLV-1 serologic studies

Samples were initially screened at the Royal Darwin Hospital (RDH) or Institut Pasteur, Paris, using the Serodia HTLV-1 particle agglutination assay (Fujirebio, Japan) and Murex HTLV-I + II test kit (Murex Diagnostics, Dartford, UK). After November 2008, HTLV-1 screening at the RDH was with the Architect rHTLV-I/II assay. HTLV-1 serostatus was then confirmed by Western blot (HTLV Blot 2.4, MP Diagnostics) according to the kit manufacturer’s criteria at the National Serological Reference Laboratory (NRL), Melbourne, or Institut Pasteur, Paris. Attempts were made to confirm HTLV-1 infection for subjects with indeterminate Western blot results using HTLV-1 polymerase chain reaction (PCR) at the NRL. Primers and probes were designed to target a highly conserved 88 bp fragment of the *gag* gene in the p19 coding region of the Australo-Melanesian HTLV-1 subtype C. The sequence of the forward primer was AGT TCG GAG CTC AGG TCG AGA, the reverse primer was AGC AAG CAG GGT CAG GCA AAG and the probe was [6FAM]-GTCCGGCGCTCCCTTAGAGCC-[BHQ1] labeled with fluorophor FAM and Black Hole Quencher 1.

### Statistics

All analyses were performed using Stata software version 13.0 (StataCorp, Texas, USA). Comparison of patient characteristics between HTLV-1 infected and HTLV-1 uninfected subjects was performed using chi-squared tests of association, independent t-tests, or Mann-Whitney tests as appropriate. Seropositivity rates among Indigenous patients according to age and gender were calculated using the proportion of patients that tested positive for HTLV-1 within each category. Logistic regression with age category, gender and age category X gender was used to assess the influence of age on the difference in seropositivity rates according to gender. The independent associations between patient demographics and the odds of HTLV-1 infection were assessed using binary logistic regression. In order to assess whether the effects of STI were similar for the 3 different patient groups in our cohort we included a patient group X interaction term in the logistic regression model for HTLV-1 status. The interaction term was non-significant and the associations between STI and HTLV-1 status were similar across the 3 groups. We therefore included all patients in a single model and included a term for group to adjust for the small differences in HTLV-1 prevalence across groups.

The proportion of seroconversions amongst those adults tested twice was calculated as a proportion with binomial 95 % confidence intervals. Repeat testing for HTLV-1 infection was ordered when clinically indicated by the treating physician and not as part of any scheduled clinical monitoring program. Duration of follow-up in each case was from the date of the initial negative serological test to the date on which a positive test was recorded.

## Results

The HTLV-1 serostatus was known for a total of 1889 patients of whom 1254 were HTLV-1 seronegative and 635 were HTLV-1 infected (HTLV-1 Western blot positive, 629; HTLV-1 Western blot indeterminate/HTLV-1 PCR positive, 6) (Fig. 2).

### Risk factors for HTLV-1 infection

**Fig. 3:**
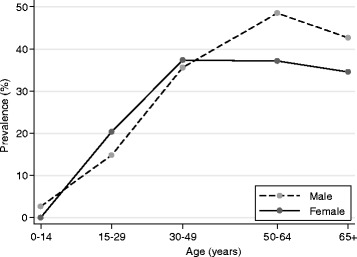
Graph of HTLV-1 seropositivity rates for Indigenous males and females according to age among 1889 patients tested at Alice Springs Hospital

Patient demographics are detailed in Table 1. Infection rates increased with age. Among 77 children who were tested, one three year old boy was HTLV-1 infected. However, only 29 of these children were from remote communities in the west or south where risk of HTLV-1 infection was highest. The HTLV-1 infected child was from the western quadrant (1 of 16 children tested, 6.25 %) and was confirmed HTLV-1 seroseropositive after being admitted with intestinal strongyloidiasis. Four of 41 adolescents (9.8 %) aged 15–17 years were also HTLV-1 infected (male, 2; female, 2), including a 15 year old from a remote community in the western quadrant who had been admitted in early childhood with complicated strongyloidiasis and was subsequently found to be HTLV-1 infected when he presented with respiratory failure complicating multi-lobar bronchiectasis. In contrast, 611 of 1703 (35.9 %) adult residents of central Australia were HTLV-1 seropositive. Seropositivity rates among all adults continued to increase with age (Fig. 3) (15–29 years, 17.3 %; 30–49 years, 36.2 %; 50–64 years, 41.7 %). Rates were similar between genders overall (Table 1), however, seropositivity rates for men older than 49 years were significantly higher than those for women (men 50–64 years 48.5 % versus women 50–64 years, 37.2 %; men >65 years, 42.7 % versus women >65 years, 34.6 %) (*p* = 0.04 for age category-by-gender interaction) (Fig. 3).

**Table 1:** Demographics and patient characteristics for 1889 Indigenous patients admitted 2000-2013

	No HTLV-1 (*N* = 1,254)	HTLV-1 (*N* = 635)	*p*-value
Age, years (±SD)	40.9 ± 17.3	47.4 ± 13.8	<0.001
Gender, n (%)			
Female	670 (53.4)	327 (51.5)	
Male	584 (46.6)	308 (48.5)	0.43
Residence, n (%)			
Remote^a^	652 (52.0)	389 (61.3)	<0.001
Town Camp^b^	193 (15.4)	131 (20.6)	
Urban^c^	206 (16.4)	70 (11.0)	
Nursing Home	42 (3.4)	22 (3.5)	
Tennant Creek	132 (10.5)	13 (2.0)	
Outside region^d^	23 (1.8)	9 (1.4)	
Missing^e^	6 (0.5)	1 (0.2)	
Sexually Transmitted Infections^f^, n (%)			
Syphilis	198 (38.6)	167 (59.4)	<0.001
Tested	513	281	
Gonorrhea	78 (18.9)	40 (15.9)	0.33
Tested	413	251	
Chlamydia	51 (12.7)	24 (10.0)	0.31
Tested	401	240	
Died, n (%)	270 (21.5)	163 (25.7)	0.04
Age at death, years (±SD)	53.0 ± 14.0	54.7 ± 13.3	0.21
Length of follow-up, years (±SD)	4.2 ± 2.9	4.6 ± 2.9	<0.001

^a^Residence in a remote community >80 km from Alice Springs, but not in the township of Tennant Creek

^b^Residence in a town camp in the Alice Springs township

^c^Residence in the Alice Springs township, but not in a town camp

^d^Residence outside the combined areas of central Australia and the adjacent Aboriginal lands of South Australia and Western Australia

^e^Place of residence could not be ascertained

^f^Any positive test during the study period among subjects aged 15–45 years who were tested

Rates were lowest among residents of remote communities north of Alice Springs (12.6 %) and highest among those from communities to the south (61.1 %) and west (42.2 %). When compared according to residence type, seropositivity rates were highest among town camp residents of Alice Springs (40.4 %) (Table 1). In a multivariable model, risk of HTLV-1 infection was significantly reduced among urban residents of Alice Springs and residents of Tennant Creek, a town in the Barkly region of the Northern Territory (Fig 1), and increased among remote residents of communities to the south and west of Alice Springs (Table 2). In contrast, male gender and a ten year increase in age independently increased the odds of infection by approximately 40 % each (male gender, *p* = 0.01; age, *p* < 0.001). Men aged 50–64 years were twice more likely than women of similar age to be HTLV-1 infected after adjusting for region, gender and STI history (OR = 2.4 (0.95, 6.18; *p* = 0.06).

**Table 2:** Multivariable predictors of HTLV-1 infection among 1889 Indigenous patients

		Model 1^a^			Model 2^a^	
	Odds ratio	95 % CI	*p*-value	Odds ratio	95 % CI	*p*-value
Age (years)	1.03	1.02–1.04	<0.001	1.04	1.04–1.03	<0.001
Gender (0 = F,1 = M)	1.18	0.97–1.45	0.10	1.42	1.09–1.86	0.01
Residence^b^						
Remote^c^	1.00					
Town Camp^d^	1.04	0.80–1.36	0.77			
Urban^e^	0.59	0.43–0.80	0.001			
N/H	0.56	0.32–0.98	0.04			
Tennant Creek	0.17	0.09–0.31	<0.001			
Outside region^f^	0.65	0.29–1.45	0.29			
Residence region^g^						
North				1.00		
East				1.53	0.8–2.8	0.17
South				10.7	7.3–15.5	<0.001
West				4.4	3.1–6.2	<0.001
STI^h^	1.93	1.53–2.45	<0.001	1.42	1.04–1.97	0.036
Patient Group STI interaction^i^						
Group 1 (*n* = 1431)	1.00			1.00		
Group 2 (*n* = 334)	0.80	0.60–1.05	0.11	0.99	0.71–1.39	0.95
Group 3 (*n* = 124)	0.89	0.60–1.34	0.59	0.71	0.39–1.28	0.26

*Abbreviations*: *N/H* residence in a nursing home, *STI* sexually transmitted infections

^a^The independent effects of residence and residence region were assessed separately in order to avoid posssible colinearity

^b^Including 1882 subjects whose place of residence was known

^c^Residence in a remote community >80 km from Alice Springs, but not in the township of Tennant Creek

^d^Residence in a town camp in the Alice Springs township

^e^Residence in Alice Springs township, but not in a town camp

^f^Residence outside the combined areas of central Australia and the adjacent Aboriginal lands of South Australia and Western Australia

^g^Including 1233 subjects who resided in remote communities categorized according to quadrants relative to Alice Springs

^h^Any positive test during the study period among subjects aged 15–45 years who were tested

^i^Reasons for HTLV-1 testing included: a) to investigate the cause of conditions thought to be HTLV-1 associated (Group 1, *n* = 1431), b) as part of a blood borne virus surveillance program among patients receiving haemodialysis (Group 2, *n* = 334) and c) after enrollment as subjects without current clinical evidence of HTLV-1 associated conditions in HTLV-1 pathogenesis studies (Group 3, *n* = 124). To demonstrate that these groups can be combined for the purposes of analysis, we determined whether there was a difference in the association between HTLV-1 and STI across groups using a Group x STI interaction term. This was non-significant, indicating that the estimated association with STI is the same across all groups, which were therefore combined for analysis. Data for HTLV-1 serostatus according to age and gender for each group is presented in Table 3

**Table 3:** HTLV-1 seropositivity rates stratified by age and gender according to reason for testing

	Age categories (years) HTLV-1+/n (%)				
	0–14	15–29	30–49	50–64	65+
Female					
Group 1 (*n* = 760)	0/36 (0.0)	32/150 (21.3)	125/341 (36.7)	63/157 (40.1)	30/76 (39.5)
Group 2 (*n* = 173)	0	1/7 (14.3)	22/64 (34.4)	23/79 (29.1)	6/23 (26.1)
Group 3 (*n* = 64)	0	0/7 (0.0)	13/26 (50.0)	11/22 (50.0)	1/9 (11.1)
Male					
Group 1 (*n* = 671)	1/37 (2.7)	16/114 (14.0)	132/341 (38.7)	48/117 (41.0)	29/62 (46.8)
Group 2 (*n* = 161)	0	2/9 (22.2)	20/77 (26.0)	39/64 (60.9)	4/11 (36.4)
Group 3 (*n* = 60)	0/1 (0.0)	1/10 (10.0)	6/28 (21.4)	9/17 (52.9)	1/4 (25.0)

HTLV-1 seropositivity rates for the various age categories for male and female Indigenous patients according to reason for testing. Subjects were tested: a) to investigate the cause of conditions that are thought to be associated with HTLV-1 infection (Group 1, *n* = 1431), b) as part of a blood borne virus (BBV) surveillance program among patients receiving haemodialysis (Group 2, *n* = 334) and c) after enrollment as subjects without current clinical evidence of HTLV-1 associated conditions in HTLV-1 pathogenesis studies (Group 3, *n* = 124). Seropositivity rates increase with age for both males and females irrespective of the reason for testing and in each group rates among men aged greater than 65 years exceed those for women of comparable age

There were no differences in HTLV-1 seropositivity rates according to the reason for testing (Table 3). Seropositivity rates for Groups 1, 2 and 3 were 33.3 % (476/1431), 35.0 % (117/334) and 33.9 % (42/124), respectively (*p* = 0.826). Similarly, there was no difference in rates of infection between these groups after adjusting for age and gender.

### Other sexually transmitted infections

There were no differences between HTLV-1 seropositive and seronegative groups in the proportion of patients who tested positive for *C.trachomatis* and *N.gonorrhoeae* NAAT (Table 1). However, syphilis serology was more often positive among those who were HTLV-1 infected (HTLV-1 seropositive, 59.4 %; HTLV-1 seronegative, 38.8 %) (*p* < 0.001) (Table 1) and having previously had an STI was significantly associated with HTLV-1 infection in multivariable models (Table 2).

### Horizontal transmission

Among 351 adults who were tested more than once during a total of 1231 years at risk and under observation, two Indigenous women and one man (3/351, 0.85 %) acquired HTLV-1 infection. The seroconversion rate in this sub-group was therefore 0.24 per 100 person-years (95 % CI = 0.18–2.48). Serodia PA were negative for both women at ages 29 and 51 years, but positive results were subsequently recorded and confirmed by Western blot at 37 and 53 years, respectively. The man was HTLV-1 seronegative by serodia PA when 62 years old. A positive result was recorded two years later at which time a Western blot was indeterminate. He was finally confirmed to be HTLV-1 infected by HTLV-1 PCR at age 65 years. A review of medical records for each case failed to reveal iatrogenic risk factors for acquiring HTLV-1 infection, such as haemodialysis or a history of blood transfusion.

### Estimated infection risk among infants

Seropositivity rates among women of childbearing age (15–40 years) varied according to their place of residence relative to Alice Springs (Table 4). The highest rate was found among women residing in the southern quadrant (49 %), the lowest among those from the northern quadrant (7 %) and the mean for all quadrants was 23 %. During a period of three years from 2010–2012, 1289 Indigenous women delivered 1351 live infants at ASH (mean (SD), 456 ± 19 infants per year). The number of Indigenous infants who were potentially at risk of mother-to-child HTLV-1 transmission was therefore 297 over the three years for which data was available (Table 4).

**Table 4:** Estimated numbers of infants born to HTLV-1 infected mothers, 2010-2012

Maternal residence	Live infants	HTLV-1 infected women (15–40 years)		Infants at risk^b^
		WB+/tested^a^	%	
Remote^c^				
North	273	6/88	7	19
East	98	3/22	14	14
South	167	26/53	49	82
West	260	22/99	23	60
Alice Springs^d^	422	38/132	29	122
Tennant Creek^e^	131	0/21	0	0
Total^f^	1351	95/415	23	297

*Abbreviations*: *HTLV-1*, infection with the Human T-Lymphotropic Virus type 1, *WB+* western blot positive

^a^The number of women aged 15–40 years who tested positive for HTLV-1 infection divided by the number tested

^b^The number of infants at risk of mother-to-child HTLV-1 infection was calculated by multiplying the estimated number of HTLV-1 infected women aged 15–40 years by the number of live infants whose mothers resided in each location

^c^Residence in a remote community according to quadrant relative to Alice Springs

^d^Any residence in the Alice Springs township

^e^Residence in the township of Tennant Creek

^f^Excluding 3 infants born to women from outside the region and 1 whose place of residence could not be ascertained

## Discussion

In a large hospital-based cohort that included nearly 13 % of Indigenous adult residents of central Australia, 35.9 % of adults tested were HTLV-1 infected, corresponding to a minimum period prevalence of 4.6 % based on 2006 regional population estimates for central Australia and the adjacent APY lands of South Australia (13,305 adults) [17]. We also demonstrate considerable variability in HTLV-1 seropositivity according to place of residence, suggesting that rates may be substantially higher in some communities. Micro-geographic variation in prevalence is a feature of HTLV-1 epidemiology elsewhere [2]. In south-western Japan, for example, seroprevalence rates exceed 30 % among adults in some villages, but are less than 10 % in those nearby [18]. The virus is likely to be similarly concentrated within some isolated communities in central Australia where HTLV-1c is thought to have been present for ~9,000 years [3]. The HTLV-1 endemic area in Australia is vast, extending from the far north of Western Australia [6] to communities in South Australia some 1000 km away [5] and covering the entire ASH catchment area of 1 million km^2^ [5, 16].

Consistent with studies in other endemic areas [1, 15, 18–23], the risk of HTLV-1 infection among Indigenous Australians increased substantially with age. Elsewhere, rates among women are typically higher than those for men due to a gender-based difference in sexual transmission that is thought to result from the close cell association of the virus [18, 19]. Thus, incidence rates for men and women living in discordant heterosexual relationships are 1.2 and 4.9 per 100 person-years, respectively [24]. Infection rates among women increase with age and are typically higher than those for men, presumably reflecting a heightened risk of transmission that accompanies physiological changes in the female genital tract [25]. In contrast, central Australian men were more likely to be HTLV-1 infected than women of similar age. An increased risk of HTLV-1 infection among men relative to women has not been described previously; however, ornamental scarification increases risk of HTLV-1 infection in Guinea-Bissau [26]. Similar cultural practices in central Australia have largely been confined to men in the context of initiation rites, which may have resulted in blood contact between participants in the past [27]. Modifications to these practices and the introduction of single-use blades may have contributed to a cohort effect among men in the present study.

Sexual transmission is also likely in central Australia. Higher numbers of sexual partners [28–32] and a longer sexual relationship with a partner at risk of HTLV-1 infection [33] predict HTLV-1 infection. Incidence rates of HTLV-1 infection in other HTLV-1 endemic areas are therefore higher among commercial sex workers (0.7 per 100 person years) [34] and subjects attending sexual health clinics (0.8–0.9 per 100 person-years) [29, 35] relative to the general public (0.09–0.19 per 100 person-years) [36–38]. The seroconversion rate recorded by us for a subset of our adult cohort (0.24 per 100 person-years) was therefore somewhat higher than incidence rates in the general public elsewhere. However, we were unable to repeat HTLV-1 serological tests for most subjects and this seroconversion rate may not reflect the true HTLV-1 incidence rate among Indigenous adults in central Australia. The association between STI history and HTLV-1 infection reported here is supported by previous studies. The risk of acquiring HTLV-1 infection is enhanced by the presence of genital lesions [39] and STIs, including syphilis [29, 39] and gonorrhea [29]. High regional STI incidence rates [40, 41] are therefore likely to increase the risk of sexually acquired HTLV-1 infection in central Australia.

We also document childhood infection, which is likely to be responsible for the cases of ATLL [4] and infective dermatitis [8] that have recently been reported from central Australia. Early childhood infection typically results from the continued exposure of infants to HTLV-1 infected cells in breast milk [15] after the loss of protective maternal antibodies, which occurs at a mean postnatal age of eleven months [42]. Consequently, infection rates increase substantially with the duration of breast-feeding; 3.9–7.4 % at 6–7 months, [15, 42–45]; 14.4–20.3 % after 6 months [15, 43, 45] and 32 % if breast feeding is continued longer than 12 months [42]. Indigenous Australian children are often breast-fed for several years [46], long after the loss of protective maternal antibodies. In resource-limited settings, weaning within 6 months has been advocated as a means of reducing risk of mother-to-child HTLV-1 transmission [43, 47, 48]. Although this approach has been criticized due to concerns that infection-related infant mortality may be increased in resource limited countries [49], incidence rates have decreased where breast-feeding by HTLV-1 infected mothers has been restricted in other settings. In Japan, for example, HTLV-1 infected mothers are advised not to breast feed [15, 50] or to do so for <3 months [50], a strategy that has been accompanied by a marked fall in the incidence rates of HTLV-1 infection. The safe implementation of a program to prevent mother-to-child transmission of HTLV-1 by providing women with appropriate education, infant formula and lactation inhibitors has also recently been reported from Brazil [51]. In contrast, Government agencies that are responsible for public health in Australia have not introduced any coordinated strategy to reduce the risk of HTLV-1 transmission to Indigenous children. HTLV-1 testing is not included in routine antenatal screening in central Australia [13] and there is no health literacy program by which Indigenous mothers can be informed of the potential risks posed by HTLV-1 infection to their children.

The retrospective design and hospital-based setting for this study results in a number of limitations. Firstly, our data is derived from a large cohort of hospitalized subjects for whom HTLV-1 serology was most often requested for clinical reasons. The resulting seropositivity rates are therefore subject to selection bias and community-based prevalence may be lower. The absence of any difference in HTLV-1 seropositivity between patients tested for clinical reasons and those who were recruited as asymptomatic controls is likely to reflect the fact that most HTLV-1 tests at ASH are ordered to accompany strongyloides serology [5]. The purpose of this practice is to identify HTLV-1/*S.stercoralis* coinfected subjects who require closer follow-up. We have previously demonstrated that only 36 % of such patients are HTLV-1 infected and that strongyloides seropositivity is not associated with HTLV-1 serostatus [5], presumably because the risk of exposure to *S.stercoralis* in communities with poor sanitation is independent of HTLV-1 infection. Selection bias is also likely to affect our estimate of the number of infants at risk because this was extrapolated from seropositivity rates for hospitalized women of childbearing age, which may over-estimate community-based HTLV-1 prevalence in this group. The number of children at risk of mother-to-child HTLV-1 transmission may therefore be less than our estimate of 297 children over three years or 99 children per year. We are also unable to determine the modes of HTLV-1 transmission for childhood infection or for those adults whose seroconversion was documented later in life. However, the literature suggests that vertical transmission is most likely to account for the former [15, 42, 43] and sexual transmission for the latter [1, 19, 29, 33].

## Conclusion

In a hospital-based cohort, we report childhood HTLV-1 infection that is suggestive of mother-to-child transmission, an increased risk of infection among older men that may reflect past cultural practices and document horizontal transmission that is likely to result from sexual contact. Community-based epidemiological studies are now needed to determine the true HTLV-1 prevalence and to define the relative contributions of the various modes of transmission. However, our findings suggest that multiple modes of transmission are likely to contribute to the high prevalence of HTLV-1 infection among Indigenous Australians. The development of any intervention to control HTLV-1 transmission therefore requires careful community engagement, cultural understanding and Indigenous leadership. High seropositivity rates [5], significant morbidity [4, 5] and evidence of sexual transmission also argue for the inclusion of this virus in the National Aboriginal and Torres Strait Islander BBV and STI Strategy [52]. Unfortunately, in the quarter-century since HTLV-1 was first shown to be endemic to central Australia, there has been no systematic attempt to provide Indigenous Australians with information about this infection. Developing the necessary health literacy required to implement any strategy to prevent HTLV-1 transmission therefore remains a considerable challenge.
